# Low Genetic Diversity and Structuring of the *Arapaima* (Osteoglossiformes, Arapaimidae) Population of the Araguaia-Tocantins Basin

**DOI:** 10.3389/fgene.2017.00159

**Published:** 2017-10-24

**Authors:** Carla A. Vitorino, Fabrícia Nogueira, Issakar L. Souza, Juliana Araripe, Paulo C. Venere

**Affiliations:** ^1^Instituto de Biociências, Universidade Federal de Mato Grosso, Cuiabá, Brazil; ^2^Instituto de Estudos Costeiros, Universidade Federal do Pará, Bragança, Brazil; ^3^Departamento de Biologia Celular, Embriologia e Genética, Universidade Federal de Santa Catarina, Florianópolis, Brazil

**Keywords:** genetic structure, microsatellite, conservation, habitat fragmentation, genetics population

## Abstract

The arapaima, *Arapaima gigas*, is a fish whose populations are threatened by both overfishing and the ongoing destruction of its natural habitats. In the Amazon basin, varying levels of population structure have been found in *A. gigas*, although no data are available on the genetic diversity or structure of the populations found in the Araguaia-Tocantins basin, which has a topographic profile, hydrological regime, and history of fishing quite distinct from those of the Amazon. In this context, microsatellite markers were used to assess the genetic diversity and connectivity of five wild *A. gigas* populations in the Araguaia-Tocantins basin. The results of the analysis indicated low levels of genetic diversity in comparison with other *A. gigas* populations, studied in the Amazon basin. The AMOVA revealed that the *Arapaima* populations of the Araguaia-Tocantins basin are structured significantly. No correlation was found between pairwise *F_ST_* values and the geographical distance among populations. The low level of genetic variability and the evidence of restricted gene flow may both be accounted for by overfishing, as well as the other human impacts that these populations have been exposed to over the years. The genetic fragility of these populations demands attention, given that future environmental changes (natural or otherwise) may further reduce these indices and eventually endanger these populations. The results of this study emphasize the need to take the genetic differences among the study populations into account when planning management measures and conservation strategies for the arapaima stocks of the Araguaia-Tocantins basin.

## Introduction

Worldwide, the populations of many fish species are declining rapidly (Allan et al., 2005), with the communities occupying lakes, rivers and floodplains being the most affected (Abell et al., 2008). In the Neotropical region, the biodiversity of aquatic ecosystems is under intense pressure, primarily from human activities, such as overfishing, pollution, habitat fragmentation, deforestation and the introduction of exotic species (Agostinho et al., 2005).

The arapaima, *Arapaima gigas* (Schinz, 1822), is a fish of considerable economic importance in the neotropics, and is one of the species listed in the Convention of International Trade in Endangered Species of Wild Fauna and Flora II. The species is listed as “Data Deficient” by the IUCN (World Conservation Monitoring Centre, 2014). As the arapaima prefers the lentic environments, such as flooded forests, rivers and lakes, of the Amazon, Araguaia-Tocantins and Essequibo basins (Castello and Stewart, 2010; Castello et al., 2013), its populations become increasingly concentrated during the dry season, and the high densities of fish that accumulate during this period greatly increase their vulnerability to capture. Given this vulnerability of the species to fishing and the reduction of its stocks in recent years, researchers have focused increasingly on its genetic diversity and population structure (Farias et al., 2003; Hrbek et al., 2005, 2007; Hrbek and Farias, 2008; Hamoy et al., 2008; Araripe et al., 2013; Vitorino et al., 2015), chromosomal evolution (Marques et al., 2006), and other aspects of its biology (Gomes, 2007; Castello, 2008a,b; Arantes et al., 2010; Fernandes et al., 2012; Farias et al., 2015).

Despite this recent interest, many features of the biology of arapaima are still relatively poorly known, in particular the structure of its natural populations. Up until now, in addition, most studies have focused on the populations of the Amazon basin, and little is known about the genetic diversity of the populations that inhabit the Araguaia-Tocantins basin, even though this system is considered to be a priority area for the conservation of the aquatic biodiversity of the Cerrado biome.

As the characteristics of the Araguaia-Tocantins basin are quite distinct from those of the Amazon basin, the data available for Amazonian *Arapaima gigas* cannot be extrapolated reliably to the Araguaia-Tocantins. In this way, Latrubesse and Stevaux (2002) identified three principal topographical sectors in the Araguaia basin (the principal focus of the present study): (i) the upper Araguaia River, which extends from the headwaters to Registro do Araguaia, located mainly on Pre-Cambrian rocks, with a V-shaped valley and many rapids, (ii) the middle Araguaia River, between Registro do Araguaia and Conceição do Araguaia, which is characterized by a well-developed alluvial plain located on the lowland Bananal Plain, a prominent geomorphological and sedimentary unit, with some isolated rock formations along the main channel, which form small rapids, and (iii) the lower Araguaia River, which runs downstream from the Bananal Plain, over an area of crystalline rocks until its confluence with Tocantins River, which has no well-defined alluvial plain (Latrubesse and Stevaux, 2002).

Because of these characteristics, the hydrological regime of the Araguaia River is also quite unique, and is determined by well-defined dry and rainy seasons (Aquino et al., 2008). The flood pulse is extremely rapid, with floodplain lakes being connected for only short periods of time. The Araguaia-Tocantins basin also includes portions of the two principal Brazilian biomes, the Amazon, to the north and the Cerrado, to the south (Aquino et al., 2005). However, these important centers of biodiversity have been impacted intensively by industrial-scale farming operations and extensive cattle ranching, the construction of reservoirs for the production of hydroelectric energy (Aquino et al., 2008, 2009; Latrubesse et al., 2009), and the establishment of the Tocantins-Araguaia waterway (Almeida and Peres, 2007). All these impacts have resulted in extensive alterations of the region’s natural environments, in particular the fragmentation of habitats, which reduces gene flow among populations through processes such as siltation and the restriction of aquatic environments (Agostinho et al., 2005).

**FIGURE 1:**
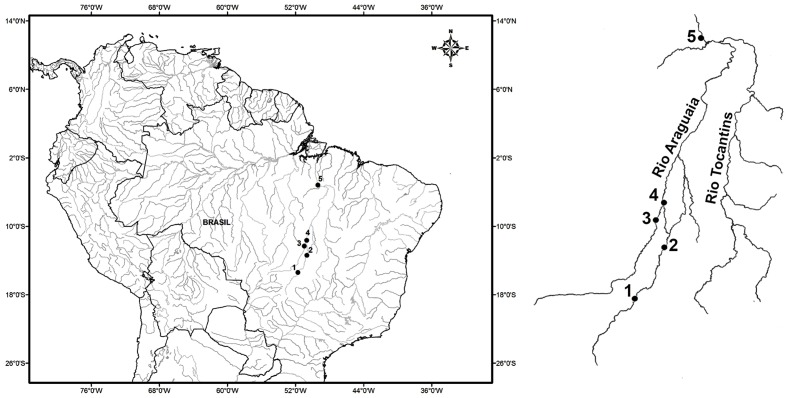
Map of Brazil, with emphasis on the Araguaia-Tocantins basin, indicating the collection sites of *Arapaima gigas*: (1) Araguaiana-MT, (2) Luís Alves, (3) Novo Santo Antônio-MT, (4) São Felix do Araguaia-MT and (5) Itupiranga-PA.

While arapaima stocks have been reduced by both fishing and habitat loss, few populations have been studied in the Araguaia-Tocantins basin, and there is a pressing need for the expansion of the database to provide a reliable assessment of the conservation status of these populations. The first study to use genetic markers in arapaima from the Araguaia-Tocantins basin was conducted by Vitorino et al. (2015), who found low levels of expected heterozygosity and a distinct structure among the four study populations. The present study provides further advances in the understanding of the genetic characteristics of this important species of Neotropical fish, through the investigation of wild arapaima populations in the Araguaia-Tocantins basin using microsatellite markers. The present study aimed to confirm the findings of Vitorino et al. (2015), and in particular, determine the existence of significant structuring in the arapaima populations of this basin. The prediction that significant population structure exists within the study area is based on the observation that, under natural conditions, arapaima typically form relatively stable family groups, which migrate laterally over short distances (Castello and Stewart, 2010; Castello et al., 2013). In this context, habitat structure is also expected to contribute to genetic diversity, given that marginal lakes increase in number and size moving downstream from the sampling point furthest upstream (point 1, **Figure 1**), with a larger number of environments been expected to support larger numbers of fish and family groups.

While the study of Vitorino et al. (2015) analyzed the genetic diversity of the arapaima populations of the Araguaia-Tocantins basin using ISSR markers, microsatellites are more appropriate for population analyses, given their co-dominant inheritance (vs. dominant alleles in the ISSR markers) and high degree of polymorphism. Microsatellite markers thus provide a much greater potential for statistical analyses, and were expected to provide a detailed database for the more reliable definition of the genetic diversity of populations. These analyses have important implications for the definition of priority areas for conservation and the implementation of management units for this species.

## Materials and Methods

Two hundred and ninety-four samples of the muscle tissue and fins of *Arapaima gigas* were obtained from five different locations on the Araguaia and Tocantins rivers, three in the state of Mato Grosso (Araguaiana, Novo Santo Antônio, and São Félix do Araguaia), one in Goiás (Luiz Alves), and one in Pará, Itupiranga (**Figure 1** and **Table 1**). The municipality of Araguaiana represents the region furthest upstream of the middle Araguaia River, where the river first forms marginal lakes along its course, appropriate for *A. gigas*. Araguaiana is also the sector with the smallest number of lakes (Alves and Carvalho, 2007). Luis Alves is just upstream from the bifurcation in the river that creates the Javaés River, and forms Bananal Island. It is important to note that this sector of the river is very popular with sport fisherman, due to the large number of lakes found in this area (Alves and Carvalho, 2007). Novo Santo Antônio, a municipality located between the Mortes and Araguaia rivers, is the region’s principal arapaima fishery center, and the lakes found along the course of the Mortes River provide the bulk of the catch landed on the Araguaia River (Kirsten et al., 2012). The municipality of São Félix do Araguaia is located at the confluence of the Mortes and Araguaia river, on the Bananal plain (de Melo et al., 2007). Most of the arapaima fishermen of Mato Grosso are resident in this municipality (Kirsten et al., 2012). The municipality of Itupiranga is located downstream of the confluence of Araguaia with Tocantins River, 170 km upstream from the Tucurui hydroelectric dam.

**Table 1:** Localization and number of individual (*n*) of Arapaima analyzed in this study.

Locality	Coordinates	*n*
Araguaiana – MT	15°23′58″S/51°43′00″W	60
Luís Alves – GO	13°22′361″S/50°40′08″W	76
Novo Santo Antônio – MT	12°18′56″S/50°58′24″W	86
São Félix do Araguaia – MT	11°38′26″S/50°40′43″W	50
Itupiranga – PA	5°10′46″S/49°21′44″W	14


The samples collected in Araguaiana were obtained during a rescue operation, which translocated fish from seasonal lakes to a perennial body of water. Small pieces of fin were collected from the live fish during this operation. All the other tissue samples were obtained from specimens caught and marketed by local fishermen at each site, so no specimens were euthanized specifically for the purposes of the study. All the samples (fin and muscle) were preserved in 100% alcohol, and deposited at the Cytogenetics and Animal Genetics Laboratory, of the Federal University of Mato Grosso (LabGen/GEPEMA/UFMT) in Cuiabá, Brazil. When it was necessary to handle animals, all procedures adhered to the recommendations of the Guide for the Care and Use of Laboratory Animals. The collection and transportation of biological specimens by the authors of the present study is authorized by the Brazilian Institute for the Environment and Renewable Resources (IBAMA) through permanent license number 15226-1, issued by the Chico Mendes Institute for the conservation of Biodiversity (ICMBio).

The total DNA was extracted according to the saline extraction protocol of Aljanabi and Martinez (1997), with minor modifications. The amount and quality of the DNA obtained through this procedure were analyzed in a Biophotometer Plus (Eppendorf Hamburg, Hamburg, Germany), and the samples were diluted to a final concentration of 5 ng/μL.

Seven primer pairs (AgCTm4, AgCTm7, AgCAm2, AgCAm15, AgCAm16, AgCAm20, and AgCAm26) were selected based on previous studies and also because they are highly polymorphic for the *Arapaima* populations of the Amazon Basin (Farias et al., 2003, 2015; Araripe et al., 2013). These primers fluorescently labeled were used for amplification of the microsatellite regions by Polymerase Chain Reaction (PCR), following the conditions described by Farias et al. (2003). For genotyping, the PCR product was mixed with a standard molecular weight marker (MegaBACE ET-550R Size Standard), which was injected into a MegaBace 1000 automatic sequencer (Amersham Biosciences). The alleles were identified using Fragment Profiler 1.2 (Amersham Biosciences).

Once the database was assembled, the existence of possible genotyping errors, null alleles or scoring was verified in MicroChecker (Van Oosterhout et al., 2004). To test whether the populations were in Hardy-Weinberg equilibrium, Genepop (Raymond and Rousset, 1995) was used to estimate the intrapopulation fixation index or coefficient of inbreeding (*F_IS_*) for each locus, which was compared with the null hypothesis (*F_IS_* = 0). Genepop was also used to determine allele frequencies, and the presence of polymorphic loci, and exclusive and rare alleles. Allele richness was estimated in Fstat 2.9.3.2 (Goudet, 2002). Expected and observed heterozygosity were determined by Arlequin 3.5.1.2 (Excoffier and Lischer, 2010). The significance of the differences between expected and observed heterozygosity was evaluated using a one-way ANOVA, run in PAST 2.17c (Hammer et al., 2001).

Genetic differentiation among populations was evaluated using an Analysis of Molecular Variance (AMOVA), the molecular fixation index (*F_ST_*), and the divergence parameter (*R_ST_*), which were all determined by Arlequin 3.5.1.2 (Excoffier and Lischer, 2010). A Mantel test was used to verify possible correlation between genetic differentiation (*F_ST_*, *R_ST_* and *F_ST_*/1-*F_ST_*) and geographical distance, which was measured following the main channel of the river.

The probability of a given number of stocks was based on a Bayesian approach run in STRUCTURE 2.3.3 (Pritchard et al., 2000). The number of presumed populations (K) was set from 1 to 7. The analyses had a burn-in of 100,000 runs, and a Monte Carlo Markov Chain (MCMC) of 1,000,000, using a model without admixture and allele frequencies. The number of populations was defined by the delta *k*-value, obtained using the STRUCTURE HARVESTER program (Earl and VonHoldt, 2012).

The estimated effective size (N_e_) of each population was derived from the theta values (θ) generated in MIGRATE-n 3.2.6 (Beerli and Felsenstein, 2001), using the formula N_e_ = θ/4μ, with a microsatellite mutation rate (μ) of 5.56 × 10^-4^ per locus per generation (Whittaker et al., 2003; Yue et al., 2007).

The MIGRATE-n program was also used to calculate migration rates between pairs of populations by the coalescence method. The number of migrants per generation (Nm) was obtained by multiplying the migration rates obtained by the program (*M* = m/μ, where m is the fraction of new immigrants from the population per generation) by the θ values of the receptor population in each pairwise comparison. This analysis was performed using the Brownian model, with a uniform distribution and a constant mutation rate between the loci. The standard search strategy values of the MIGRATE program were used, except for the use of 20 short chains and 5 long chains.

The BOTTLENECK program, version 1.2.02 (Cornuet and Luikart, 1996) was used to verify the existence of recent demographic events, such as population bottlenecks. The Wilcoxon test was run using the Two-Phased Mutation (T.P.M) model, established with 30% for the Infinite Allele Mutation (IAM) model and 70% for the Stepwise Mutation Model (SMM).

## Results

### Genetic Diversity

A total of 25 alleles were identified, with an average of 3.57 alleles per locus. While all the loci were polymorphic, four were monomorphic in at least one of the arapaima populations analyzed from the Araguaia-Tocantins basin. The number of alleles, observed and expected heterozygosity, allelic richness, and the fixation index (*F_IS_*) were calculated for all seven microsatellite loci analyzed, together with the mean parameters for all loci (**Table 2**).

**Table 2:** Genetic diversity indexes for *Arapaima gigas*, using microsatellite markers.

Loci		Population				
		
		Ara	LAl	NSA	SFA	Itu
AgCTm7	*N*	2	2	2	2	2
	A_R_	1.999	1.993	1.997	2.000	3.000
	H_O_	0.271	0.254	0.294	0.520	0.714^∗^
	H_E_	0.305	0.346	0.284	0.504	0.679
	*Fis*	0.115	-0.168	-0.033	-0.032	-0.053^∗^
AgCAm20	*N*	1	1	1	1	3
	A_R_	1.000	1.000	1.000	1.000	3.000
	H_O_	Mono	Mono	Mono	Mono	0.428
	H_E_	Mono	Mono	Mono	Mono	0.574
	*Fis*	–	–	–	–	0.261
AgCAm2	*N*	1	3	5	4	3
	A_R_	1.000	2.247	2.676	2.913	3.000
	H_O_	Mono	0.260	0.131^∗^	0.286	0.215
	H_E_	Mono	0.236	0.188	0.253	0.315
	*Fis*	–	-0.103	0.304^∗^	0.004	0.328
AgCAm26	*N*	2	2	2	2	2
	A_R_	1.998	2.000	2.000	2.000	2.000
	H_O_	0.356	0.587	0.442	0.480	0.571
	H_E_	0.295	0.501	0.449	0.504	0.518
	*Fis*	-0.208	-0.172	0.017	0.049	-0.106
AgCAm16	*N*	2	2	2	2	1
	A_R_	1.995	1.335	1.894	1.738	1.000
	H_O_	0.271	0.026	0.094^∗^	0.0802	Mono
	H_E_	0.261	0.026	0.132	0.078	Mono
	*Fis*	-0.04	-0.006	0.288^∗^	-0.032	–
AgCTm4	*N*	1	3	3	3	2
	A_R_	1.000	2.203	2.174	2.094	2.000
	H_O_	Mono	0.123	0.059^∗^	0.120	0.286
	H_E_	Mono	0.118	0.144	0.115	0.508
	*Fis*	–	-0.04	0.592^∗^	-0.044	0.477
AgCAm15	*N*	2	2	3	3	3
	A_R_	1.689	2.000	2.171	2.280	3.000
	H_O_	0.036	0.343	0.4393	0.3203^∗^	0.786
	H_E_	0.069	0.339	0.4643	0.4793	0.553
	*Fis*	0.488	-0.011	0.055	0.359^∗^	-0.444
Mean	*N*	1.571	2.143	2.571	2.429	2.429
	A_R_	1.526	1.825	1.987	2.004	2.429
	H_O_	0.133	0.228	0.209	0.258	0.429
	H_E_	0.133	0.190	0.176	0.276	0.450
Total	P_L_	4	6	6	6	6
	Allele	11	15	18	17	17
	% allele	44%	60%	72%	68%	68%


*N, number of alleles; A_*R*_, allelic richness; H_*O*_, observed heterozygosity; H_*E*_, expected heterozygosity; *Fis*, coefficient of inbreeding computed as in Weir and Cockerham (1984); P_*L*_, polymorphic locus; –, monomorphic locus. ^∗^*P* < 0.05. Ara, Araguaiana; LAl, Luís Alves; NSA, Novo Santo Antônio; SFA, São Felix do Araguaia and Itu, Itupiranga.*

The population from Novo Santo Antônio had the highest genetic diversity, with 72% of the identified alleles and an allelic richness of 2.429. At the opposite extreme, the arapaima from Araguaiana were the least diverse (44.0%) with an allelic richness of only 1.521. The specimens from Araguaiana also returned the lowest heterozygosity (Ho = 0.133, He = 0.133), while those from Itupiranga had the highest values, with observed heterozygosity of 0.428 and an expected heterozygosity of 0.449 (**Table 2**).

Five loci deviated from Hardy-Weinberg equilibrium, with heterozygote frequencies being either lower or higher than expected (**Table 2**). Only four of the 28 values estimated for the inbreeding coefficient (*F_IS_*) were significantly positive, which indicates a deficit of heterozygotes. Three of these loci were recorded in the population from Novo Santo Antônio, while the other was from São Felix do Araguaia. A single locus from Itupiranga was significantly negative, indicating an excess of heterozygotes. When the whole set of loci is considered, a significant *F_IS_* value (*p* < 0.001) was only for the Novo Santo Antônio presented, indicating the occurrence of inbreeding in this population.

One to three alleles, with high frequencies, were recorded for most loci. All loci except AgCAm15 presented exclusive alleles for the different study populations, at frequencies ranging from 0.012 to 1.00. No exclusive alleles was detected in the population from São Felix do Araguaia (**Table 3**). No new alleles were detected in this study, given that all the alleles recorded here had been reported previously by Farias et al. (2003) and Araripe (2008).

**Table 3:** Allele frequency and presence of exclusive alleles.

Loci	Allele	Population				
		
		Ara	LAl	NSA	SFA	Itu
AgCTm7	279	0.186	0.851	0.171	0.520	0.250
	285	0.814	0.148	0.829	0.480	0.357
	295	0.000	0.000	0.000	0.000	0.393^∗^
AgCAm20	265	0.000	0.000	0.000	0.000	0.393^∗^
	267	0.000	0.000	0.000	0.000	0.071^∗^
	269	1.000	1.000	1.000	1.000	0.536
AgCAm2	313	0.000	0.000	0.006	0.000	0.821
	315	0.000	0.000	0.000	0.010	0.036
	317	1.000^∗^	0.000	0.000	0.000	0.000
	321	0.000	0.055	0.012	0.0,31	0.000
	323	0.000	0.868	0.899	0.837	0.143
	325	0.000	0.076	0.071	0.122	0.000
	333	0.000	0.000	0.012^∗^	0.000	0.000
AgCAm26	213	0.822	0.532	0.663	0.520	0.500
	217	0.178	0.468	0.337	0.480	0.500
AgCAm16	251	0.153^∗^	0.000	0.000	0.000	0.000
	255	0.000	0.986	0.929	0.960	1.000
	257	0.847	0.013	0.071	0.040	0.000
AgCTm4	275	0.000	0.041^∗^	0.000	0.000	0.000
	277	1.000	0.938	0.924	0.940	0.571
	279	0.000	0.020	0.065	0.050	0.000
	283	0.000	0.000	0.012	0.010	0.429
AgCAm15	228	0.036	0.785	0.646	0.650	0.464
	230	0.000	0.000	0.006	0.010	0.036
	244	0.964	0.214	0.348	0.340	0.500


*Ara, Araguaiana; LAl, Luís Alves; NSA, Novo Santo Antônio; SFA, São Félix do Araguaia and Itu, Itupiranga. ^∗^Exclusive allele.*

The MicroChecker analysis (**Table 3**) indicated the presence of null alleles in the populations from Luís Alves (for loci AgCAm20 and AgCTm4), Novo Santo Antônio (loci AgCAm2, AgCTm4, AgCAm16 and AgCAm20), São Félix do Araguaia (AgCAm15), and Itupiranga (AgCTm4). No systematic pattern was observed in the occurrence of null alleles in the different study populations, however. No evidence was found of either the misidentification of stutters as alleles or dropouts (dominance of small alleles).

### Population Structure

**FIGURE 2:**
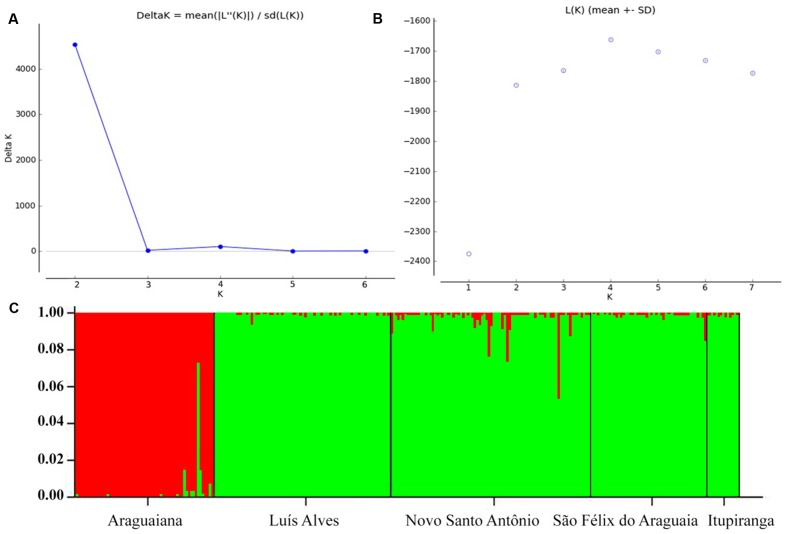
Genetic structuring of *Arapaima gigas* from the Araguaia-Tocantins basin, according to Bayesian analysis. Estimating the number of K groups based on **(A)** ΔK and **(B)** the mean maximum likelihood. **(C)** Each column represents a different individual and the colors represent genetic stocks. The collection sites are separated by black lines.

The values recorded for Wright index (*F_ST_*) ranged from 0.061 to 0.669, while those for the Slatkin divergence parameter (*R_ST_*), varied from 0.025 to 0.688. Both indices were significant for all pairs of locations (**Table 4**). The results of the Mantel test rejected the hypothesis that genetic differentiation was related to geographical distance, considering the values of either *F_ST_* (*r*^2^ = 0.466115, *p* = 0.236), *R_ST_* (*r*^2^ = 0.452354, *p* = 0.240) or *F_ST_*/1-*F_ST_* (*r*^2^ = 0.219112, *p* = 0.221000). The results of the Bayesian inference based on the mean likelihood [Ln (K)] of the Δk (Evanno et al., 2005) indicate the presence of two clusters within the Araguaia-Tocantins basin, one formed by the populations of Luís Alves, Novo Santo Antônio, São Félix do Araguaia, and Itupiranga, and the other by that of Araguaiana, which forms a distinct group (**Figure 2**). These findings were used to establish the clusters for the analysis of molecular variance (AMOVA).

**Table 4:** *F_ST_*/*R_ST_* values and geographic distance in kilometers (above the diagonal) between *A. gigas* populations from Araguaia-Tocantins basin.

Population	Ara	LAl	NSA	SFA	Itu
Ara	–	329.8	653.2	594.9	1589.3
LAl	0.669/0.688	–	323.4	265.1	1259.5
NSA	0.563/0.512	0.245/0.146	–	111	1105.4
SFA	0.577/0.548	0.061/0.044	0.072/00251	–	994.4
Itu	0.600/0.4384	0.425/0.510	0.396/0.3224	0.322/0.3431	–


*Ara, Araguaiana; LAl, Luís Alves; NSA, Novo Santo Antônio; SFA, São Félix do Araguaia and Itu, Itupiranga.*

The results of the AMOVA indicated a lack of significant variation between the two groups (Φ*_ST_* = 44.15%, *R_ST_* = 40.07%). The variation among populations within each group contributed only 12.35% (Φ*_ST_*) and 11.17% (*R_ST_*) of the variance, while the variance within populations is 43.51% for Φ*_ST_* and 48.76 for *R_ST_*. The Φ*_ST_* (Φ*_ST_* = 0.564; *p* = 0.0000) and (*R_ST_* = 0.512; *p* = 0.000) were both relatively high among the study populations.

Effective population size ranged from 128 to 351 individuals, with the lowest value being recorded at Araguaiana and the highest at São Félix do Araguaia (**Table 5**). The MIGRATE-n coalescence analysis (Beerli and Felsenstein, 2001) indicated low levels of gene flow between populations, and only six of the 20 estimates of migrant numbers were higher than one migrant per generation.

**Table 5:** Analysis of the MIGRATE program showing the estimates of gene flow peer-to-peer among the Arapaima populations of the Araguaia-Tocantins region.

Emigrant population	θ	N_e_	Nm [Receiving population]				
			
	(4N_e_μ)	(θ/4μ)	Ara	LAl	NSA	SFA	Itu
Ara	0.2847	128.0126	-	0.7053	0.8615	0.8624	0.3151
	(0.258–0.315)	(116.0–141.63)		(0.444–1.074)	(0.543–1.308)	(0.549–1.306)	(0.184–0.546)
LAl	0.5284	237.5899	0.0632	–	0.5272	1.5895	0.2170
	(0.485–0.576)	(218.07–258.99)	(0.023–0.139)		(0.295–0.875)	(1.117–2.220)	(0.116–0.400)
NSA	0.5573	250.5845	0.3663	1.1346	–	1.2733	0.0904
	(0.516–0.602)	(232.01–270.68)	(0.234–0.553)	(0.776–1.618)		(0.860–1.831)	(0.036–0.203)
SFA	0.7820	351.6187	0.3529	1.0905	2.7187	–	0.1386
	(0.713–0.860)	(320.59–386.69)	(0.225–0.534)	(0.742–1,559)	(2.039–3.575)		(0.065–0.280)
Itu	0.4237	190.5126	0.3330	1.2991	0.6836	0.1635	–
	(0.362–0.524)	(162.76–235.61)	(0.210–0.507)	(0.905–1.821)	(0.379–1.102)	(0.061–0.357)	


*The same pair of populations may have different numbers of immigrant and migrant individuals. Ln(L): -2473.954. θ = Population genetic parameter defined as 4N_*e*_μ (Effective population size multiplied by the mutation rate), Ne, estimate of effective population size. Nm, estimate of the number of migrants defined as NeM (effective population size multiplied by the migration rate). Ln (L) = Ln(L), Natural log of the probability of the estimated parameters. Ara, Araguaiana; LAl, Luís Alves; NSA, Novo Santo Antônio; SFA, São Félix do Araguaia and Itu, Itupiranga.*

The Bottleneck program did not detect any significant deficit of heterozygotes. However, a significant (*p* < 0.05) excess of heterozygotes was found in the Itupiranga population using the TPM (Wilcoxon test) model.

## Discussion

This study is the first to use microsatellite markers to examine the genetic diversity of the arapaima populations of the Araguaia-Tocantins basin. Overall, the results indicated significantly lower levels of genetic diversity and heterozygosity than those found in previous studies of the same genetic markers in populations from other areas (Farias et al., 2003, 2015; Hamoy et al., 2008; de Alencar Leão, 2009; Araripe et al., 2013).

The arapaima populations that inhabit the study region are affected by natural processes, in particular the hydrological regime, that are quite distinct in comparison with the populations found in the Amazon basin, the region in which the species has been investigated in most detail (Vitorino et al., 2015). The unique features of the Araguaia-Tocantins basin may influence the reduced genetic diversity (number of alleles, heterozygosity, and allelic richness) of its population in comparison with those from the Amazonian basin. This is also true of the allelic richness of the arapaima populations of Tucuruí (Araripe, 2008) and Bananal Island (de Alencar Leão, 2009), two other areas located within the Araguaia-Tocantins basin, which present low values in comparison with population from the Amazon basin. The levels of genetic diversity recorded in the *Arapaima* populations of the Araguaia-Tocantins basin are lower than expected for the species, but are consistent with the findings of Vitorino et al. (2015), based on ISSR markers. Using mitochondrial markers, da Penha (2014) also found relatively low genetic variability in arapaima populations of the Araguaia-Tocantins basin in comparison with those of the Amazon basin.

The points sampled in the present study are exploited intensively by local fisheries. In fact, most of the arapaima marketed locally are harvested from natural populations (Çiftci and Okumus, 2002), including not only adults, but also the capture of fry as stock for rearing on fish farms. This type of overexploitation often results in a decline in effective population size (N_e_), provoking the loss of genetic diversity, which leads to a reduced resilience of populations to environmental stresses and climate change, and a loss of resistance to pathogens (Allendorf and Luikart, 2007; Hughes et al., 2015).

The variation in the N_e_ values estimated for the different populations analyzed in the present study indicates potential differences in their population dynamics, with distinct patterns of fluctuation over time in the number of individuals reproducing, a process that will have knock-on effects for the sustainability of the population (Carson et al., 2011). In other words, each arapaima population may have a distinct demographic history, based on the differences in variables such as the number of mature individuals, the sex ratio, offspring survival rates, and the availability and quality of habitats. However, the results of the Bottleneck analysis indicate a significant impact on genetic structure only in the case of the population from Itupiranga. This may be related to the construction of the hydroelectric dam at Tucuruí, and the creation of one of the world’s largest reservoirs, which caused considerable impacts on the local fish fauna of the Tocantins River.

The reduced genetic variability found in the arapaima populations of the Araguaia-Tocantins basin may also be related to the anthropogenic impacts that have altered the natural features of the basin extensively throughout most of its length. The hydrological cycle of this basin is more intense than that of the Amazon, and this cycle has been modified by local farming and ranching activities, and the establishment of the Araguaia-Tocantins waterway (Latrubesse and Stevaux, 2006). These modifications have had a major impact, principally on the region’s lacustrine environments, causing a reduction in the size of its lakes, and altering their flood cycle, which affects the lateral migrations typical of this fish (Castello and Stewart, 2010; Castello et al., 2013). These changes may force the arapaima to remain in the same lakes, unable to migrate in search of reproductive partners, eventually creating small, isolated populations susceptible to inbreeding. Genetic drift tends to have a greater impact in smaller populations, which also favor inbreeding, exacerbating the loss of genetic diversity. It seems likely that the combined effects of these processes have reduced the reproductive potential of the local arapaima populations, impacting their effective size. The geomorphology and flood cycle of the Araguaia-Tocantins and Amazon basins are quite distinct, and this appears to be the principal factor determining the variation in the genetic diversity of the stocks analyzed from the Amazon (Farias et al., 2003, 2015; Hamoy et al., 2008; de Alencar Leão, 2009; Araripe et al., 2013) and the Tocantins-Araguaia basin (Vitorino et al., 2015).

However, the possibility that low genetic variability is a natural characteristic of the arapaima populations of the Araguaia-Tocantins basin cannot be ruled out altogether. But whatever the determining factors, this genetic fragility is a cause for concern, given that future environmental impacts (natural or otherwise) may further reduce the diversity of these populations, and threaten their long-term viability.

This fragility is further reinforced by the differences in the genetic variability of each population in the Araguaia-Tocantins basin. Specimens collected at Araguaiana, for example, had the lowest genetic diversity of any population, indicating the smallest effective size of any population, which implies that this population is the most vulnerable to anthropogenic interference from the expansion of the agricultural frontier occurring within the basin (Latrubesse and Stevaux, 2006), as well as being the sector of the basin that has the smallest number of lakes, the preferred habitat of the arapaima (Alves and Carvalho, 2007).

The most likely explanation for the deviations from Hardy-Weinberg equilibrium detected in the arapaima populations of the Araguaia-Tocantins basin is inbreeding, given that significant *F_IS_* values (indicating a deficiency of heterozygotes) were found in six of the eight deviations recorded. A number of studies have recorded a deficiency of heterozygotes in fish populations (Castric et al., 2002; Langen et al., 2011; O’Leary et al., 2013; Ferreira et al., 2015), indicating that inbreeding may be relatively common in these vertebrates. In the arapaima this may be related to the relatively sedentary behavior of the species and its parental care, as observed in a number of other species of Neotropical fish (Sofia et al., 2006, 2008; Ferreira et al., 2015).

In addition to these behavioral traits, arapaima is intolerant of lotic environments, so areas of strong rapids represent effective barriers to the dispersal of this species (Castello and Stewart, 2010; Castello et al., 2013). This may lead to the formation of family groups in the different micro-regions of the Araguaia-Tocantins basin. Genetic differences between subpopulations related to geographic distance or the presence of physical barriers, such as waterfalls, have been identified in Amazonian arapaima (Araripe et al., 2013) and in other fish species (Hughes et al., 2015).

The pairwise *F_ST_* values indicate moderate (0.05–0.15) to extreme (>0.25) genetic differentiation between populations. However, the Bayesian analysis points to the presence of only two genetic stocks in the Araguaia-Tocantins basin, one at Araguaiana, and the other formed by the remaining populations, at Luís Alves, Novo Santo Antônio, Sao Félix do Araguaia and Itupiranga (**Figure 2**). This arrangement contrasts with that recorded by Vitorino et al. (2015), based on ISSR markers, which indicated that the Araguaiana and Novo Santo Antônio populations shared the same genetic stock, while São Félix do Araguaia and Itupiranga form separate populations (Luís Alves was not included in this analysis).

The structure proposed by the Bayesian analysis is also supported by the estimates of gene flow and the number of migrants between populations, given that the populations that share the same genetic stock had the highest migration rates. It is important to note that the gene flow detected here may reflect ancestral processes, rather than the recent exchange of individuals, because the number of migrants is a somewhat abstract quantity, which cannot be distinguished from the effective population size (Balloux and Lugon-Moulin, 2002).

While the genetic variation found in the present study is not related systematically to geographic distance, the geographically closer populations (Luís Alves, Novo Santo Antônio and São Félix do Araguaia) returned the lowest *F_ST_* and *R_ST_* values. Reduced genetic differentiation was expected between the samples from Novo Santo Antônio and São Félix do Araguaia, which are the geographically closest sites (separated by a distance of only 111 km), although the *F_ST_* values indicate that the populations of São Félix do Araguaia and Luís Alves (265.1 km apart) are the most similar. In addition to the basic differences in comparison with the Amazonian arapaima populations, then, the relationship between population structure and geographic distance is also distinct from that recorded by Araripe et al. (2013).

The present study confirmed the low genetic diversity of the *Arapaima gigas* populations of the Araguaia-Tocantins basin, which can be linked directly to the environmental fragility of this river system, which reinforces the need for a better understanding of the processes that may further reduce the viability of these populations. The findings of this study also indicated that the genetic diversity of the populations is distributed heterogeneously within the study area, and that the establishment of a single protected area would be insufficient for the preservation of the genetic diversity of the arapaima populations of the Araguaia-Tocantins basin as a whole. In particular, future management measures should consider the population from Araguaiana as an independent unit, distinct from the other Araguaia-Tocantins populations. The Itupiranga region should also be defined as a priority area for conservation, given the high allelic richness found in this population.

## Ethics Statement

This study was carried out in strict accordance with the recommendations provided in the Guide for the Care and Use of laboratory Animals. During the development of this work, no animals were sacrificed. Collection was authorized by SEMA (license number 104358/2011/SEMA-MT), Instituto Brasileiro do Meio Ambiente e dos Recursos Naturais Renováveis - IBAMA and Instituto Chico Mendes de Conservação da Biodiversidade – ICMBio (License number 15226-1).

## Author Contributions

CV performed the molecular genetic studies, performed the statistical analyzes and drafted the manuscript. FN performed some molecular genetic studies and contributed to the correction of the text. JA and PV conceived and coordinated the study, participated in its elaboration and helped to draft the manuscript. All authors read and approved the final manuscript.

## Conflict of Interest Statement

The authors declare that the research was conducted in the absence of any commercial or financial relationships that could be construed as a potential conflict of interest.
